# Outdoor temperature, blood pressure, and cardiovascular disease mortality among 23 000 individuals with diagnosed cardiovascular diseases from China

**DOI:** 10.1093/eurheartj/ehv023

**Published:** 2015-02-17

**Authors:** Ling Yang, Liming Li, Sarah Lewington, Yu Guo, Paul Sherliker, Zheng Bian, Rory Collins, Richard Peto, Yun Liu, Rong Yang, Yongrui Zhang, Guangchun Li, Shumei Liu, Zhengming Chen

**Affiliations:** 1 Clinical Trial Service Unit and Epidemiological Studies Unit (CTSU), University of Oxford, Richard Doll Building, Old Road Campus, Oxford OX3 7LF, UK; 2 Chinese Academy of Medical Sciences, Dong Cheng District, Beijing, China; 3 Department of Epidemiology, School of Public Health,Peking University Health Science Centre, Beijing, China; 4 NCDs Prevention and Control Department, Liuzhou CDC, Liuzhou, Guangxi, China; 5 NCDs Prevention and Control Department, Pengzhou CDC, Pengzhou, Sichuan, China; 6 NCDs Prevention and Control Department, Gansu CDC, Lanzhou, Gansu, China; 7 NCDs Prevention and Control Department, Hunan CDC, Changsha, Hunan, China; 8 NCDs Prevention and Control Department, Heilongjiang CDC, Harbin, Heilongjiang, China

**Keywords:** Blood pressure, Seasonal variation, Cardiovascular disease, Hypertension, Cohort study, China

## Abstract

**Introduction:**

Blood pressure is a major cause of cardiovascular disease (CVD) and both may increase as outdoor temperatures fall. However, there are still limited data about seasonal variation in blood pressure and CVD mortality among patients with prior-CVD.

**Methods:**

We analysed data on 23 000 individuals with prior-CVD who were recruited from 10 diverse regions into the China Kadoorie Biobank during 2004–8. After 7 years of follow-up, 1484 CVD deaths were recorded. Baseline survey data were used to assess seasonal variation in systolic blood pressure (SBP) and its association with outdoor temperature. Cox regression was used to examine the association of usual SBP with subsequent CVD mortality, and seasonal variation in CVD mortality was assessed by Poisson regression. All analyses were adjusted for age, sex, and region.

**Results:**

Mean SBP was significantly higher in winter than in summer (145 vs. 136 mmHg, *P* < 0.001), especially among those without central heating. Above 5°C, each 10°C lower outdoor temperature was associated with 6.2 mmHg higher SBP. Systolic blood pressure predicted subsequent CVD mortality, with each 10 mmHg higher usual SBP associated with 21% (95% confidence interval: 16–27%) increased risk. Cardiovascular disease mortality varied by season, with 41% (21–63%) higher risk in winter compared with summer.

**Conclusion:**

Among adult Chinese with prior-CVD, there is both increased blood pressure and CVD mortality in winter. Careful monitoring and more aggressive blood pressure lowering treatment in the cold months are needed to help reduce the winter excess CVD mortality in high-risk individuals.

**See page 1152 for the editorial comment on this article (doi:10.1093/eurheartj/ehv024)**

## Introduction

Cardiovascular disease (CVD) is a leading cause of mortality and morbidity, with an estimated 17 million deaths in 2008.^1^ Over 80% of CVD deaths now occur in low- and middle-income countries such as China,^2^ and the burden of disease from CVD is projected to increase further over the next few decades in these countries.^3^

Increased blood pressure is one of the most important modifiable causes of CVD,^4–6^ and is known to be associated with lifestyle and environmental factors. There is evidence that outdoor temperature affects blood pressure,^7–9^ with particularly large effects in China.^8^ Exposure to cold temperatures can lead to vasoconstriction and tachycardia, both of which contribute to increased blood pressure and cardiac load.^10^ Excess CVD mortality has been reported during the cold seasons,^11–15^ part of which may be driven by temperature-related increases in blood pressure. Individuals who are already suffering from CVD may be at, particularly, increased risk when exposed to cold temperatures. However, limited large-scale data exist about seasonal variation in blood pressure and CVD mortality among people with prior-CVD, especially in China where few people have access to central heating in winter and where few people with prior-CVD are properly managed.^16^

We report data from the China Kadoorie Biobank (CKB) study of over 500 000 adults aged 30–79 years who were recruited from 10 diverse regions in China during 2004–8 and followed up ever since for mortality and morbidity to 31 December 2013. The present study is of 23 000 participants who reported at the baseline visit having a physician diagnosed CVD and aims (a) to examine the association of outdoor temperature with blood pressure measured at recruitment, both overall and in different population subgroups, (b) to investigate the association between usual blood pressure and subsequent CVD mortality, and (c) to assess any seasonal variation in CVD mortality rates and then compare it with predicted risk based on seasonal changes in blood pressure.

## Methods

### Baseline survey

**Figure 1:**
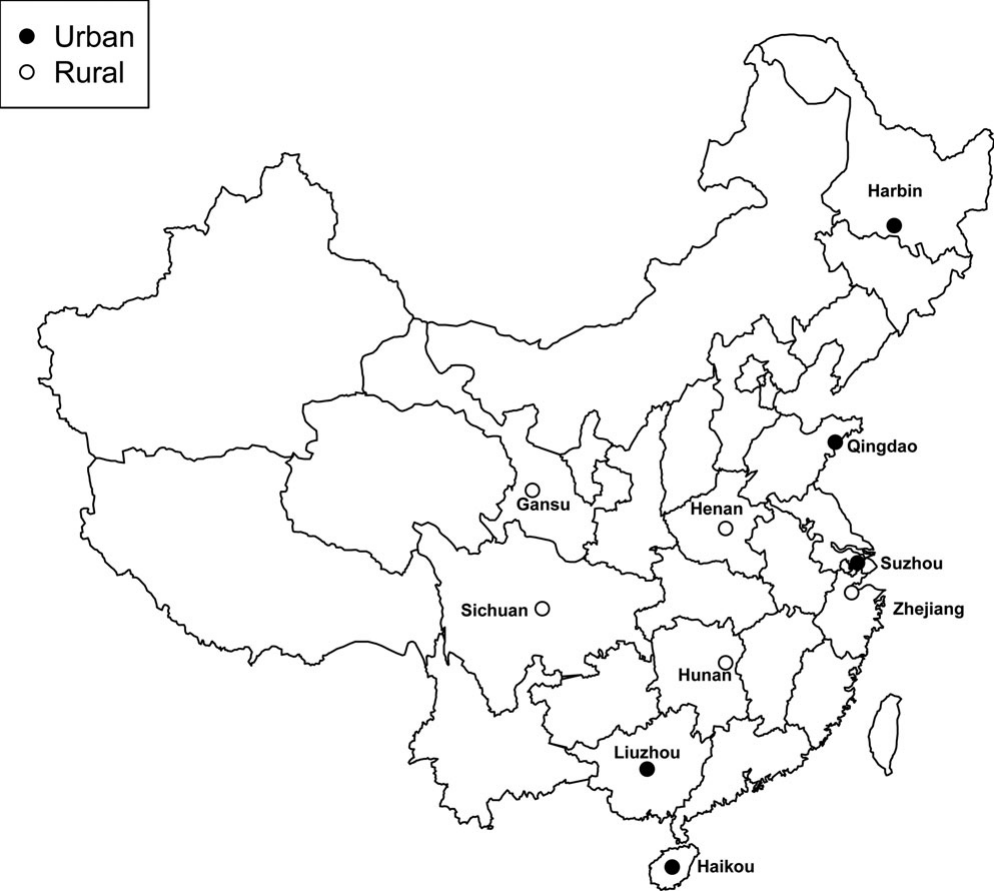
Locations of the recruitment centres in China Kadoorie Biobank. The solid circles denote urban areas, and the open circles denote rural areas.

Detailed information about the study design and procedures has been reported previously.^8,17^ Briefly, the baseline survey took place between 2004 and 2008 in 10 geographically defined areas in China (*Figure 1*). At the baseline survey, detailed information about general demographic and socio-economic status, dietary and other lifestyle habits (e.g. smoking, alcohol drinking, and physical activity), indoor air pollution, medical history, and current medication were collected using an interviewer-administered laptop-based questionnaire. Participants were asked whether they had ever been diagnosed by a physician with a range of chronic diseases [e.g. diabetes, ischaemic heart disease (IHD), stroke or transient ischaemic attack (TIA), hypertension, chronic obstructive pulmonary disease, cancer, or other common diseases), and if so, the age at first diagnosis and whether they were still on treatment. Participants who had reported having a prior history of IHD, stroke/TIA, diabetes, or hypertension were additionally asked about current usage of specific drugs (aspirin, ACE-I, β-blocker, statins, diuretics, or calcium antagonist).^16^ A range of physical measurements were undertaken for each participant and a blood sample was collected.

Ethical approval was obtained from the Ethical Review Committee of the Chinese Centre for Disease Control and Prevention, Beijing, China and the Oxford Tropical Research Ethics Committee, University of Oxford, UK. All study participants provided written informed consent.

### Blood pressure measurement

Blood pressure was measured at least twice using a UA-779 digital monitor after participants had remained at rest in a seated position for at least 5 min. If the difference between the two measurements was >10 mmHg for SBP, a third measurement was made and the last two measurements were recorded. The procedure for blood pressure measurement was standardized across the 10 study areas, and all measurements were made by trained study personnel. All devices were regularly calibrated to ensure consistency of measurements.^8,17^

### Meteorological data

Daily meteorological measurements for the years 2004–2008 were obtained from China Meteorological Administration local offices at each region. Mean monthly, summer (June, July, August), and winter (December, January, February) outdoor temperatures were calculated as the average of the recorded mean daily outdoor temperatures (the average of four measurements taken at 0200, 0800, 1400, and 2000 h) for all the participants who were surveyed during that month or season.

### Follow-up for mortality and morbidity

Since recruitment, participants have been followed up for cause-specific morbidity and mortality through linkage with regional disease and death registers and with the recently established national health insurance (HI) system. Causes of death are sought chiefly from official death certificates supplemented, if necessary, by reviewing medical records or undertaking verbal autopsy, a WHO standard tool to determine probable causes of death for those who died without any medical attention.^18^ A bespoke IT system developed by the project team for this study is used for entering the data from death and disease registers, with the death certificates or the disease reporting cards scanned. Data linkage with HI agencies is carried out every 6 months in each region, and all hospitalized events occurring in that last half-year are retrieved for matched study participants. So far, ∼98% of the study population is covered by the HI system. To minimize losses to follow-up, active follow-up (i.e. visiting local community or directly contacting participants) is also performed annually.^17^

The main analyses of the present study only involved individuals who reported having IHD, stroke/TIA at the baseline survey, i.e. defined as ‘prior-CVD participants’. For prospective analyses, only deaths from IHD (ICD-10: I20–I25) or stroke (ICD-10: I60–I61, I63–I64) were considered, henceforth referred to as ‘CVD mortality’.

### Statistical methods

Baseline characteristics—mean (SD) for continuous variables, *N* (%) for categorical variables—were calculated separately for people with or without CVD. All main analyses related to blood pressure and CVD mortality were only for prior-CVD participants. Mean systolic and diastolic blood pressure (SBP, DBP), adjusted for area, age, and sex were calculated separately for each calendar month of the study recruitment period. These blood pressure means were then plotted against the mean day of the year for each month of recruitment, i.e. regardless of the year, with January and February combined to allow for the fall in recruitment over the Chinese New Year. To obtain the estimated change in blood pressure per 10°C lower outdoor temperature, a multiple linear regression analysis was performed of individual SBP on the individual outdoor temperature, adjusted for age and sex. Observations <5°C were omitted from the regression because few participants experienced daily winter temperatures regularly well below that range, with exception of those from Harbin where the winter temperature usually drops well below −10°C and nearly all households have proper central heating from mid-October until March the following year.^8^ Subgroup analyses by area, sex, age, body mass index (BMI), education, use of anti-hypertensive treatment, medical history of hypertension or diabetes and, for men only, current smoking and alcohol drinking (there were too few female drinkers and smokers for reliable analyses) were performed to assess whether seasonal changes in blood pressure were modified by other known vascular risk factors. To help make allowance for the multiplicity of comparisons when many subgroup analyses are performed, the separate heterogeneity *χ*^2^ statistics for each were summed (as were their degrees of freedom) to yield a global test for heterogeneity.^19^ The individual heterogeneity tests are not shown where trend tests are more appropriate.

Cox proportional hazards models were used to calculate hazard ratios (HRs), with blood pressure as the exposure variable and CVD mortality as the outcome, and stratified by sex, region (10 groups), age at risk (10 groups), and anti-hypertensive treatment status, and further adjusted for smoking, alcohol drinking, BMI, and education. The 95% confidence interval (CI) for each log HR was estimated using the ‘floating absolute risk’ method, which facilitates many different comparisons and tests for trend between different categories, rather than just pair-wise comparisons between one arbitrarily chosen reference group and each of the other categories.^20^ To correct for regression dilution bias,^4,21^ HRs in the groups determined at baseline were plotted against the usual blood pressure, i.e. the mean value of systolic blood pressure in that group at the subsequent resurvey, on average 2.6 years after the baseline survey.

Finally, the number of CVD deaths and person-years for each calendar month were calculated and summed into groups by calendar month regardless of year. Poisson regression, adjusted for age, sex, and area, was used to calculate CVD mortality rates in each of the groups. Analyses were performed using SAS version 9.3 and R version 3.0.1.

## Results

A total of 506 673 people (99% of all participants) had data on both blood pressure and outdoor temperature on the day of baseline survey. *Table 1* compares the baseline characteristics and the number of deaths from CVD during follow-up between individuals with and without prior-CVD. Overall, 23 040 (4.5%) participants reported having prior-CVD with mean years since diagnosis of 7.2 years. About one-third of those with prior-CVD was from Harbin, which is located in the far northeast of China (*Tables 1* and *2*). Those with prior-CVD tended to be older (mean age of 61 vs. 51 years) and more likely to have higher mean SBP (141 vs. 131 mmHg) and BMI (24.9 vs. 23.6 kg/m^2^). At any given age, men with prior-CVD were less likely to be current regular smokers or weekly alcohol drinkers, but more likely to be ex-smokers or ex-drinkers than men without prior-CVD. The lower proportions of current regular smokers or drinkers among men with prior-CVD were probably due to increased rates of quitting because of illness. People with prior-CVD were also more likely to report a prior diagnosis of hypertension (48 vs. 10%) or diabetes (12 vs. 3%), and to be taking anti-hypertensive treatment (31 vs. 4%). During an average of 7.1 (SD 1.3) years of follow-up, a total of 1484 CVD deaths were recorded among those with prior-CVD (748 from IHD and 736 from stroke) (*Table 1*).

**Table 1:** Baseline characteristics of study participants and number of cardiovascular disease-related deaths by self-reported hospital diagnosis of prior-cardiovascular disease

	Overall	With prior-CVD	No prior-CVD
Number of participants	506 673	23 040	483 633
Age (SD, years)	52 (11)	61 (9)	51 (11)
SBP (SD, mmHg)	131 (21)	141 (23)	131 (21)
DBP (SD, mmHg)	78 (11)	80 (12)	78 (11)
BMI (SD, kg/m^2^)	23.7 (3.4)	24.9 (3.6)	23.6 (3.4)
Years since diagnosis (SD, years)		7.2 (7.1)	–
Men (%)	41.0	43.5	40.9
Current smokers (%)	61.0	43.0	61.9
Ex-smokers (%)	13.3	29.1	12.5
Weekly drinkers (%)	32.9	21.0	33.6
Ex-drinkers (%)	3.7	11.8	3.3
With central heating (%)	12.3	33.0	11.3
Self-reported hypertension (%)	11.7	48.2	10.0
Treated for hypertension (%)	4.8	30.6	3.6
Self-reported diabetes (%)	3.2	12.3	2.7
Number of IHD/stroke deaths^a^	7144	1484	5660

^a^IHD (ICD-10: I20–I25), stroke (ICD-10: I60–I61, I63–I64), follow-up duration: baseline to 31 December 2013.

**Table 2:** Mean temperature (°C) and systolic blood pressure (mmHg) in summer and winter, by area, among participants with prior-cardiovascular disease

Area	Latitude (°N)	% with central heating	Number with prior-CVDs	Summer (June–August)		Winter (December–February)		Difference (Summer vs. Winter)		Change (SE) in SBP per 10°C lower temperature^b^ (≥5°C only)
				Temp (°C)	SBP^a^	Temp (°C)	SBP^a^	Temp (°C)	SBP^a^	
Harbin	46	94	7672	22.7	131	−14.1	138	36.8	−7	5.9 (0.52)
Qingdao	36	15	2009	23.9	138	1.7	146	22.1	−9	8.3 (0.81)
Henan	35	0	3000	26.9	139	1.5	150	25.4	−12	5.4 (0.62)
Gansu	35	1	1342	22.3	139	−0.1	151	22.4	−12	9.0 (1.14)
Zhejiang	31	0	817	28.1	138	6.0	153	22.2	−15	6.9 (1.05)
Suzhou	31	2	1017	28.0	137	5.9	146	22.1	−9	5.8 (0.93)
Sichuan	31	0	476	25.7	137	6.5	144	19.2	−7	4.3 (1.55)
Hunan	28	1	2623	27.9	143	6.9	152	21.0	−9	4.8 (0.58)
Liuzhou	24	0	3423	28.7	133	12.1	144	16.5	−11	7.1 (0.59)
Haikou	20	0	661	28.6	129	18.5	140	10.2	−11	7.4 (1.87)
Overall		29	23 040	25.5	136	3.8	145	21.7	−9	6.2 (0.24)

^a^SBP, systolic blood pressure; adjusted for age, sex, and area (where appropriate).

^b^Adjusted for age, sex, and area (where appropriate).

**Figure 2:**
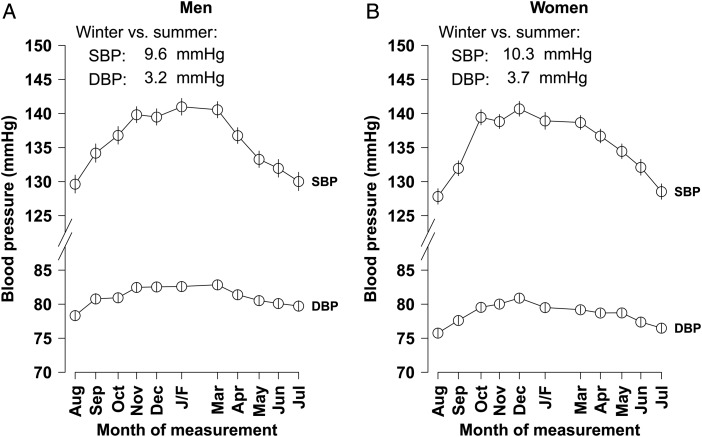
Monthly variation in blood pressure and outdoor temperature in people with prior-cardiovascular disease in (*A*) men and (*B*) women. The horizontal placement of each month combined over the full 4 years of recruitment represents the mean number of days since the first participant was recruited for participants recruited in average over years for that month. J/F = January and February combined (recruitment dropped in January and February due to the Chinese New Year). For both blood pressure and temperature, the mean monthly values are the mean for all participants whose baseline survey happened during that month (regardless of the year). Means of blood pressure were adjusted for sex and age. The winter months are placed centrally to display the winter peak in blood pressure.

**Figure 3:**
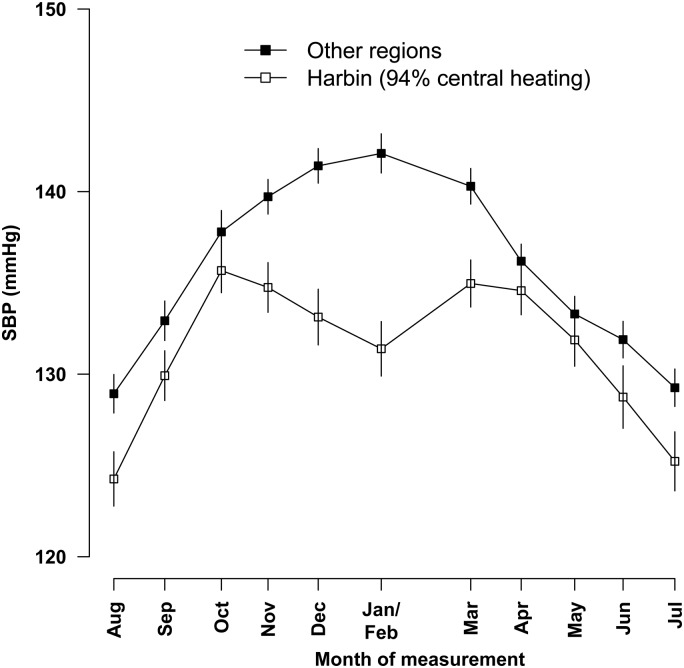
Monthly variation in systolic blood pressure and outdoor temperature among people with prior-cardiovascular disease, Other regions together vs. Harbin. Conventions as in *Figure 1*.

A seasonal cycle of mean blood pressure, especially SBP, was observed in people with prior-CVD, with highest levels in winter and lowest levels in summer in both men and women (*Figure 2*). Overall, SBP varied from 145 mmHg in winter to 136 mmHg in summer (*P* < 0.001), but the magnitude of this variation differed between regions (*Table 2*). Indeed, in Harbin, although the mean outdoor temperature was on average 36°C colder in winter than in summer, the mean SBP only differed by 7 mmHg between winter and summer seasons, with a small fall, rather than increase, in SBP in winter when the central heating was turned on (*Figure 3*).

**Figure 4:**
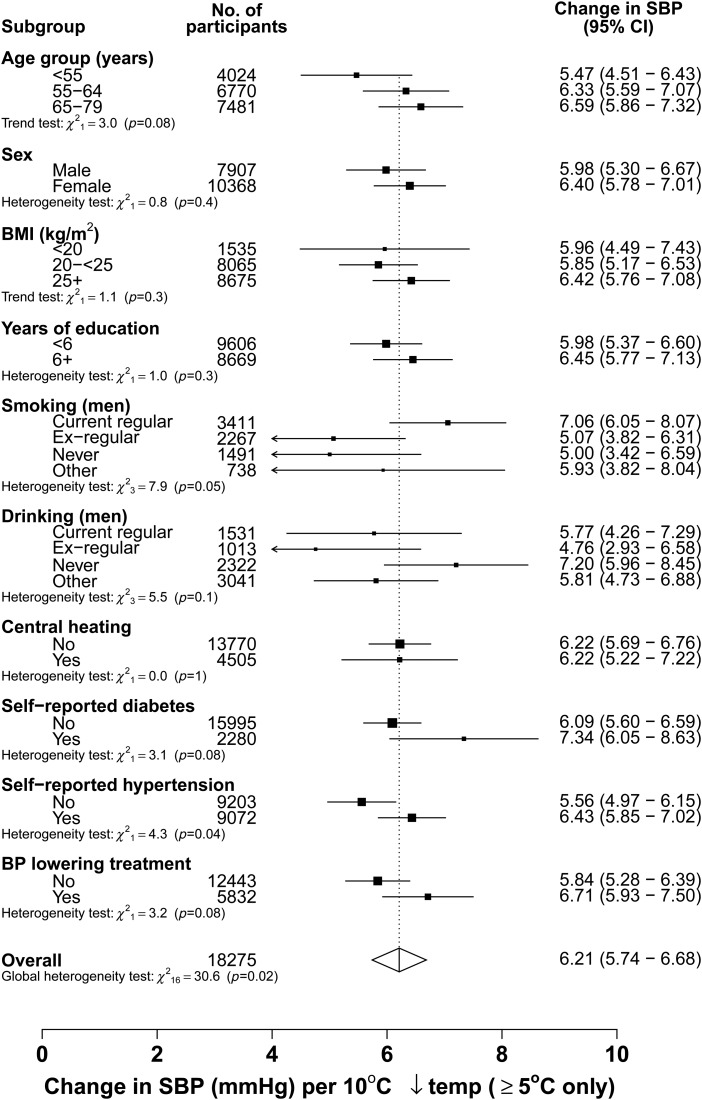
Seasonal variation in systolic blood pressure and outdoor temperature among people with prior-cardiovascular disease by various subgroups, with the temperature range at least >5°C. The analysis was adjusted for age, sex, and area (where appropriate). Each closed square represents a change in systolic blood pressure per 10°C lower outdoor temperatures. The dotted vertical line indicates the overall change in systolic blood pressure; the open diamond indicates it and its 95% CI.

There was an approximately linear inverse association between SBP and temperature >5°C, with a mean increase of 6.2 (SE 0.24) mmHg in SBP for each 10°C decrease in outdoor temperature. There was a 2-fold difference in the strength of this relationship between areas (*p*_het_ = 0.003), ranging from 4.3 mmHg in Sichuan to 9.0 mmHg in Gansu (*Table 2*, Supplementary material online, *eFigure S1*). However, the association was largely consistent between most other subgroups studied (*P* for global test of heterogeneity = 0.02) (*Figure 4*).

**Figure 5:**
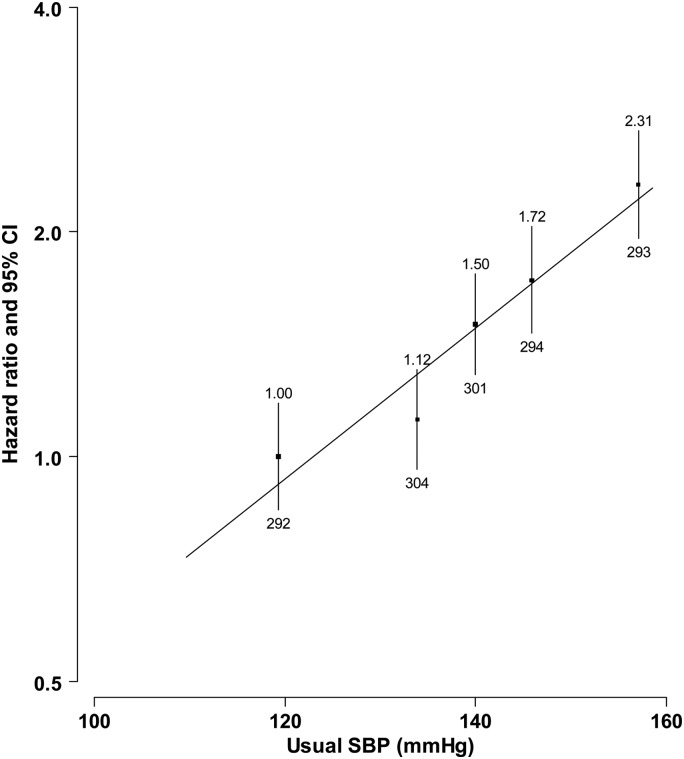
Hazard ratios for cardiovascular disease mortality vs. usual blood pressure among people with prior-cardiovascular disease. Analyses were stratified by region, age, gender, and blood pressure lowering treatment status, and adjusted for education, smoking, alcohol drinking, and body mass index. The hazard ratios are plotted on a floating absolute scale. Each square has an area inversely proportional to the standard error of the log risk. Vertical lines indicate the corresponding 95% confidence intervals. Numbers above confidence intervals are of hazard ratios and those below are the numbers of cardiovascular disease deaths.

**Figure 6:**
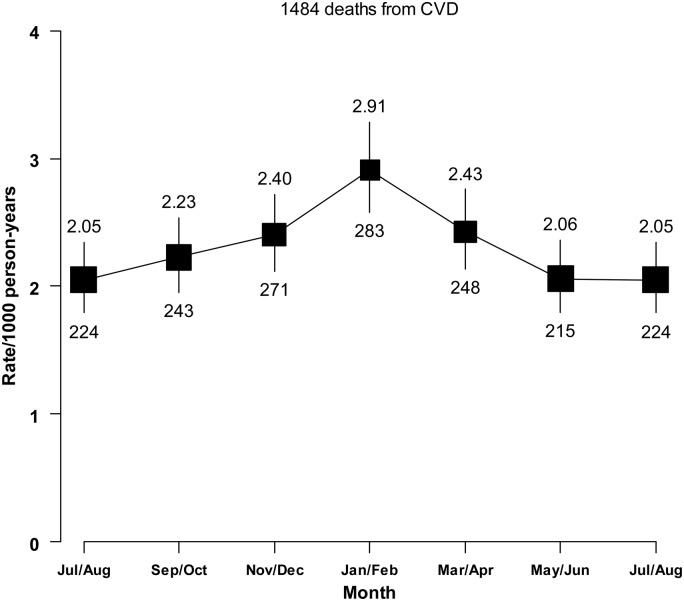
Seasonal variation in cardiovascular disease mortality rates among people with prior-cardiovascular disease, between 2004 and 2013. Deaths and person-days at risk for a given months are totalled across the follow-up period. The analysis is adjusted for age group, study site, and sex, but not for year of follow-up. To make the curve smoother, two calendar months are combined and winter months are again placed centrally, as in *Figure 1*. Vertical lines indicate the corresponding 95% confidence intervals. Numbers above confidence intervals are of mortality rates (per 1000 person-years) and those below are the numbers of deaths.

A positive association was observed between usual SBP and CVD mortality, with 21% (95% CI: 16–27%) higher risk of CVD mortality for each 10 mmHg higher usual SBP (*Figure 5*). The excess risk was similar between people treated with blood pressure lowering agents and those without (HR (95% CI) = 1.25 (1.18–1.33) vs. 1.18 (1.08–1.28), *P* for heterogeneity = 0.28). Overall, the adjusted CVD mortality rate was 2.35 per 1000 person-years, but fluctuated over the course of the year, with a winter peak observed (2.78 in winter vs. 1.98 per 1000 person-years in summer) (*Figure 6*). Among people with prior-CVD, both absolute-CVD mortality rates and the relative seasonal changes were weaker in Harbin than in the other regions (Harbin: 1.96 vs. 1.61 per 1000 person-years, rate ratio (95% CI) = 1.22 (1.07–1.39); all other combined: 4.10 vs. 2.73 per 1000 person-years, rate ratio = 1.50 (1.39–1.61)).

## Discussion

In this large study of over 23 000 individuals with prior-CVD who were recruited from the general communities in 10 diverse regions of China, we observed substantial seasonal variation of blood pressure, especially, in areas where there is little use of central heating in the cold months. Overall, the mean SBP was 9 mmHg higher in winter than in summer, and, >5°C in outdoor temperature, SBP was 6.2 mmHg higher for each 10°C decrease in temperature. Among people with prior-CVD, blood pressure is a strong independent predictor of subsequent CVD mortality, with each 10 mmHg higher usual SBP associated with ∼21% higher risk of CVD death. Mirroring the seasonal variation of blood pressure, there was a 41% increase in CVD mortality during winter.

Several studies have provided evidence about seasonal variation of blood pressure, but most of them were in general populations, with only a few involving high-risk populations such as people in old age,^7,22–24^ those with low BMI,^7,25^ hypertension,^7,24,26^ or end-stage renal disease.^27^ The present large study in China provides large-scale evidence about climate-related changes in blood pressure in people with prior-CVD. In this Chinese population, the seasonal variation in blood pressure was generally more extreme than that observed in western populations, and was abolished by the use of home central heating. The large variation in blood pressure between seasons observed in the present study has strong implications for detection and clinical management of hypertension. People maybe more likely to be diagnosed with hypertension if examined in winter than in summer, and hypertensive patients may suffer even higher blood pressure in the cold season due to inadequate blood pressure control. In a prospective study of 184 elderly Israeli patients with essential hypertension, supplementary anti-hypertensive treatment was required during winter in 38% of them.^28^ We were not able to assess seasonal changes within individuals since it is not practicable to monitor changes in participants' blood pressure and treatment during follow-up among 0.5 million participants. In our study, the mean blood pressure was much higher in winter than in summer among both those taking and those not taking blood pressure lowering treatment. Blood pressure, especially SBP, is an independent predictor of CVD risk not only in general population but also in those with prior vascular diseases.^4,29^ The beneficial effects of blood pressure lowering treatments on the risks of major CVD diseases are well established.^30^ In this study population with prior-CVD, the strength of the association – i.e. 21% higher CVD mortality per 10 mmHg higher usual SBP—was weaker than previous estimates from general populations (both Chinese and Western), that have estimated ∼40% higher stroke mortality and 30% higher IHD death.^4^ This may be explained by the fact that a high proportion of the individuals with prior-CVD in the present study were on anti-hypertensive treatment that may well attenuate the effects of blood pressure on subsequent CVD risk.

A winter peak in CVD mortality has been consistently reported among general populations in many studies.^11,12,31–35^ However, only one previous study has simultaneously examined seasonal changes in blood pressure and mortality in people with prior-CVD.^15^ In that study of 19 000 male British civil servants aged 40–69 years with 25-year follow-up , the seasonal effect on all-cause mortality was more extreme among 3284 men with prevalent IHD than those without (rate ratio for winter vs. summer of 1.38 and 1.18, respectively, *P* = 0.03). Among those with prior-IHD participants, the rate ratios were 1.31 for IHD mortality and 1.48 for stroke.^15^ In our study, where mean SBP differed by ∼10 mmHg between winter and summer, we observed 41% higher CVD mortality in winter compared with summer, which is somewhat greater than predicted from our prospective analysis of prognostic effect of blood pressure on CVD mortality, although it is comparable with the risk estimates for a prolonged 10 mmHg SBP difference reported from the general population.^4^ It is unlikely that all the epidemiologically expected risk would be observed by the short- to medium-term rise in blood pressure during winter and it is likely that there are additional factors that contributed to the winter rise in mortality. Nevertheless, some of the observed winter peak in CVD mortality may well be driven by rises in blood pressure due to the cold temperature.

There are some limitations to our study. Although it was explicit that all reported prior disease history should be based on physician diagnosis, it was not possible with such a large cohort to adjudicate all the self-reported cases retrospectively. However, there is an evidence from other studies of similar nature in China that self-reported history of CVD showed a good consistency with hospital records.^36^ Moreover, in a separate analysis when comparing the CVD mortality rates observed in our prior-CVD population with rates in those without, a 3-fold higher risk was consistently appeared in each month [overall HR = 2.98 (95% CI: 2.80–3.18)] (Supplementary material online, *eFigure S2*). This provides indirect evidence that self-reports of prior-CVD in our study were generally reliable. Occlusive and haemorrhagic CVD may involve different pathophysiological mechanisms.^37^ Although the present study included a sufficiently large number of CVD deaths to assess the overall effect of season on CVD mortality, the statistical power to separately examine the seasonal patterns of subtype of CVD mortality is rather limited. The isolated clinic BP measurements used in our study are unable to provide as reliable an assessment of blood pressure as ambulatory blood pressure monitoring, and may lead us to underestimate any patterns or associations. However, the high comparability of baseline characteristics between participants enrolled at different seasons or months, as reported previously,^8^ suggests that seasonal variation in blood pressure is likely to be driven primarily by changes in outdoor temperature rather than by other lifestyle factors.

Our study findings suggest that seasonal variation in CVD mortality rates in people with prior-CVD result, at least partly, from seasonal variation of blood pressure. Given the high prevalence of CVD in the Chinese population, especially of stroke, even moderate increases in CVD mortality rates during the winter months, as shown in this study, must account for large excess numbers of CVD deaths. The results from the present analyses suggest that seasonal changes in blood pressure should be taken into account in the diagnosis and treatment of hypertension and CVD. For patients with prior-CVD or other high-risk individuals, more intensive anti-hypertensive treatment and more frequent blood pressure monitoring may be required in winter to achieve the same blood pressure control as in other seasons. Longer follow-up of this large cohort study and the use of well-characterized non-fatal CVD events may allow for more detailed analyses of seasonal variation separately in the main components of CVD, namely IHD, ischaemic, and haemorrhagic stroke.

## Supplementary material

Supplementary material is available at *European Heart Journal* online.

## Funding

The baseline survey and the first re-survey were supported by a research grant from the Kadoorie Charitable Foundation in Hong Kong. The long-term continuation of the project during 2009–2014 is supported by program grants from the Wellcome Trust in the UK (088158/Z/09/Z) and the Chinese Ministry of Science and Technology. The UK Medical Research Council, the British Heart Foundation and Cancer Research UK also provide core funding to the Clinical Trial Service Unit and Epidemiological Studies Unit at Oxford University for the project. Funding to pay the Open Access publication charges for this article was provided by Welcome Trust.

**Conflict of interest:** none declared.

## Appendix

**Members of the China Kadoorie Biobank collaborative group**

(a) *International steering committee*

Liming Li (PI), Junshi Chen, Rory Collins, Richard Peto, Zhengming Chen (PI).

(b) *Study Coordinating Centres*

International Co-ordinating Centre, Oxford: Zhengming Chen, Garry Lancaster, Xiaoming Yang, Alex Williams, Margaret Smith, Ling Yang, Yumei Chang, Iona Millwood, Yiping Chen, Sarah Lewington.

National Co-ordinating Centre, Beijing: Yu Guo, Zheng Bian, Can Hou, Yunlong Tan, Huiyan Zhou.

Regional Co-ordinating Centres, 10 areas in China:

Qingdao

Qingdao CDC: Zengchang Pang, Shaojie Wang, Yun Zhang, Kui Zhang.

Licang CDC: Silu Liu, Wei Hou.

Heilongjiang

Provincial CDC: Zhonghou Zhao, Shumei Liu, Zhigang Pang. Nangang CDC: Weijia Feng, Shuling Wu, Liqiu Yang, Huili Han, Hui He, Bo Yu.

Hainan

Provincial CDC: Xianhai Pan, Shanqing Wang, Hongmei Wang. Meilan CDC: Xinhua Hao, Chunxing Chen, Shuxiong Lin, Xiangyang Zheng.

Jiangsu

Provincial CDC: Xiaoshu Hu, Minghao Zhou, Ming Wu, Ran Tao. Suzhou CDC: Yeyuan Wang, Yihe Hu, Liangcai Ma, Renxian Zhou, Guanqun Xu, Yan Lu.

Guangxi

Provincial CDC: Baiqing Dong, Naying Chen, Ying Huang. Liuzhou CDC: Mingqiang Li, Jinhuai Meng, Zhigao Gan, Jiujiu Xu, Yun Liu, Jingxin Qing.

Sichuan

Provincial CDC: Xianping Wu, Yali Gao, Ningmei Zhang Pengzhou CDC: Guojin Luo, Xiangsan Que, Xiaofang Chen.

Gansu

Provincial CDC: Pengfei Ge, Jian He, Xiaolan Ren. Maiji CDC: Hui Zhang, Enke Mao, Guanzhong Li, Zhongxiao Li, Jun He, Yulong Lei, Xiaoping Wang.

Henan

Provincial CDC: Guohua Liu, Baoyu Zhu, Gang Zhou, Shixian Feng. Huixian CDC: Yulian Gao, Tianyou He, Li Jiang, Jianhua Qin, Huarong Sun.

Zhejiang

Provincial CDC: Liqun Liu, Min Yu, Yaping Chen, Ruying Hu. Tongxiang CDC: Zhixiang Hu, Jianjin Hu, Yijian Qian, Zhiying Wu, Chunmei Wang, Lingli Chen.

Hunan

Provincial CDC: Wen Liu, Guangchun Li, Huilin Liu. Liuyang CDC: Xiangquan Long, Xin Xu, Youping Xiong, Zhongwen Tan, Xuqiu Xie, Yunfang Peng, Weifang Jia.
